# Clinical effectiveness of internet-based cognitive behavioral therapy for insomnia in routine secondary care: results of a randomized controlled trial

**DOI:** 10.3389/fpsyt.2024.1301489

**Published:** 2024-05-10

**Authors:** Polina Pchelina, Mikhail Poluektov, Tobias Krieger, Simone B. Duss, Thomas Berger

**Affiliations:** ^1^Department of Neurology and Neurosurgery, I.M. Sechenov First Moscow State Medical University, Moscow, Russia; ^2^Department of Clinical Psychology and Psychotherapy, Institute of Psychology, University of Bern, Bern, Switzerland; ^3^Interdisciplinary Sleep-Wake-Epilepsy-Center and Swiss Sleep House Bern, Inselspital, Bern University Hospital, University of Bern, Bern, Switzerland

**Keywords:** chronic insomnia, internet-based cognitive behavioral therapy for insomnia, Insomnia Severity Index, sensitivity analysis, care as usual

## Abstract

**Introduction:**

Delivering cognitive behavioral therapy for insomnia over the internet bears the advantage of accessibility and uptake to many patients suffering from chronic insomnia. In the current study, we aimed to investigate the effectiveness of internet-based cognitive behavioral therapy for insomnia (iCBT-I) in routine care.

**Materials and methods:**

We conducted a two-arm non-blinded randomized controlled trial with care as usual (CAU) as a control condition. Participants were recruited in a specialized outpatient sleep medicine department. Both arms had access to other healthcare resources, and the intervention group had access to the iCBT-I program for 2 months. The primary outcome was insomnia severity, measured by the Insomnia Severity Index (ISI). Secondary outcomes were fatigue severity, daytime sleepiness, affective symptoms, dysfunctional beliefs and attitudes about sleep, sleep locus of control, sleep hygiene, sleep efficiency (SE), sleep onset latency, wake time after sleep onset (WASO), and total sleep time (TST). Linear mixed models for repeated measures were used to analyze the longitudinal data at baseline, post-treatment, and after 3 months of follow-up. The trial was registered at www.clinicaltrials.gov (NCT04300218 21.04.2020).

**Results:**

The results showed a significant time*group interaction effect (*p* = 0.001) at post-treatment with between-group effect size (*d* = 0.51), indicating that the ISI decreased by a score of 3.8-fold in the iCBT-I group than in the CAU group. There was no significant difference in ISI between groups at follow-up. Regarding secondary outcomes, dysfunctional beliefs about sleep, SE, and WASO decreased significantly during treatment in the intervention group with between-group effect sizes *d* = 0.35, *d* = *−*0.51, and *d* = 0.47, respectively. At the follow-up, between-group effects on DBAS and SE remained significant: *d* = 0.36 and *d* = *−*0.63, respectively. For TST, we observed a significant time*group effect of *d* = *−*0.38 only after follow-up.

**Conclusion:**

Our findings suggest that iCBT-I has a significant effect on insomnia severity at post-treatment compared to CAU. iCBT-I further improved dysfunctional beliefs about sleep and improved subjective sleep characteristics, such as SE, WASO, and TST during 3 months after treatment.

**Clinical trial registration:**

www.clinicaltrials.gov, identifier (NCT04300218).

## 1 Introduction

Chronic insomnia (CI) is the most common sleep disorder that affects 10% of the global population (1, 2). This disorder is characterized by both night and daytime symptoms and can affect an individual’s physical, emotional, and cognitive wellbeing and impact their social functioning, and work productivity (3). Moreover, insomnia is associated with other socially significant diseases: cardiovascular and metabolic diseases, depression, and anxiety, and can be integrated into their models of pathogenesis (4–8). Cognitive behavioral therapy for insomnia (CBT-I) has emerged as the gold standard for treating CI, which is supported by international recommendations (9–11). It is a non-pharmacological, multicomponent approach that aims to modify dysfunctional sleep-related behavior and thinking patterns by means of cognitive and behavioral techniques. Although CBT-I is highly effective, there is limited access to specialized clinicians (12–14).

Internet-based cognitive behavioral therapy for insomnia (iCBT-I) has the potential to overcome these barriers (15). In this approach, the CBT-I sleep interventions are delivered to the patients through smartphone applications and online platforms, enabling greater accessibility and convenience. Prior studies have demonstrated the effectiveness of iCBT-I in improving sleep outcomes in research settings, in which participants were recruited via advertisements, online postings, and flyers and interviewed online or via telephone (16–18). However, current findings may not be generalizable to patients in routine practice, for example, due to the higher heterogeneity of patients in routine care than in research samples. Understanding iCBT-I effects in clinical settings would also help to determine its place in a stepped-care model of CBT-I delivery (19). The stepped-care model is a healthcare approach providing insomnia patients with access to the various formats of CBT-I, which currently place iCBT-I at the bottom at a preclinical level (20). The only study examining iCBT-I in a routine care setting was published by Sato et al. and was conducted at an outpatient clinic focusing on the effectiveness of iCBT-I as an adjunct to usual care in insomnia patients who remained symptomatic after pharmacotherapy. Even in this treatment-resistant sample, iCBT-I significantly improved sleep quality and subjective sleep characteristics (21). However, the question about the applicability of iCBT-I in a routine clinical population remains open. Thus, the primary aim of the study was to evaluate the clinical effectiveness of the program of the internet-based cognitive behavioral therapy for insomnia “Sleepsy” (iCBT-I) in comparison with care as usual (CAU) among patients with CI recruited from clinical settings. As a secondary aim, we evaluated the effectiveness of this program regarding affective and wellbeing symptoms, subjective sleep characteristics, sleep-related behavior, and beliefs.

## 2 Materials and methods

This was a parallel-group add-on superiority randomized controlled trial comparing an active treatment condition (iCBT-I plus CAU) to CAU alone. Interventions in CAU could be assigned within the first visit to a referring doctor or at any point of the study on the next doctor visit. Thus, participants in both groups had access to any other healthcare resources at all forms and levels of healthcare, including other forms of CBT-I during their study participation. A choice in favor of CAU as a control condition was made to (1) reproduce clinical settings and evaluate the superiority of the investigated method over routine care; and (2) to overcome ethical problems that arise when a patient with insomnia receives no treatment (22). The disadvantage of this approach is the narrowing of differences in outcome between groups, which could result in a loss of power to detect a meaningful difference (22, 23).

Inclusion criteria were age between 18 and 80 years, fulfilling the criteria of CI according to the International Classification of Sleep Disorders-3 (ICSD-3), the ability to follow the procedures of the study, fluent in Russian language, and having access to stable internet that we assessed during the first visit with the semi-structured interview elaborated for this study. Patients were excluded if they had severe depressive or anxiety symptoms or were earlier diagnosed with psychiatric comorbidities other than depression and anxiety, and if they had other sleep, neurological, or somatic disorders affecting night sleep. The presence and severity of depressive or anxiety symptoms were assessed with the Beck Anxiety Inventory (BAI), the Beck Depression Inventory-II (BDI), and other exclusion criteria, with the semi-structured interview during the first visit. After considering inclusion and non-inclusion criteria and signing the informed consent form, the participants were registered on the Qualtrics Survey platform by the study personnel and received their individual allocation code. On completion of baseline measures, subjects were automatically assigned by the randomization function of the Qualtrics Survey Software using single block randomization with a 1:1 allocation ratio to either the iCBT-I + CAU or the CAU group. Neither the participants nor the referring specialist nor the research team have foreknowledge and/or control over randomization and the allocation procedure. The participants were stratified by the clinical center to avoid a situation when participants from one regional center were allocated to the same group. Neither participants nor the study personnel were blind to treatment allocation and randomization outcomes. The detailed protocol of the study recruitment, inclusion and exclusion criteria, intervention description, and outcomes have been previously reported (24). The trial was registered at www.clinicaltrials.gov (NCT04300218) and was approved by the local ethics committee of the I.M. Sechenov Moscow Medical University (No. 03-20/19.02.2020).

The internet-based intervention was an 8-week guided iCBT-I program. The program consisted of a sleep diary and eight 10- to 15-min video lectures about homeostatic and circadian mechanisms of human sleep regulation, pathogenesis of CI, and its daytime consequences. Every next video lecture became available given that participants submitted at least two sleep diary entries during the preceding week. Video lectures provide the rationale for the psychoeducational, behavioral (bedtime restriction, stimulus control, and relaxation), and cognitive (cognitive restructuring) techniques. Within the sleep restriction technique, the patients were recommended to reduce time in bed to average self-reported sleep time over 1 week of sleep diary plus 30 min, with a minimum of 6 h and getting up at the same time every day, regardless of sleep duration. If the sleep diary indicated a sleep efficiency (SE) of 85% or higher for the previous week, the participant will be encouraged to add 15 min to the sleep window. If it did not reach 85%, the recommended sleep window was decreased based on the preferred wake-up time and average total sleep time (TST) over the period of observation. If the SE fell between 80% and 85%, the sleep window remained stable. In accordance with the stimulus control technique, participants were recommended not to go to bed unless sleepy and to not stay in bed unless asleep within 20 min. Within the relaxation techniques module, the participants were offered to try the progressive muscle relaxation autotraining for 30 min before bedtime. The cognitive techniques include dysfunctional belief restructuring (e.g., targeting unrealistic beliefs about sleep and the consequences of sleep loss).

**Figure 1:**
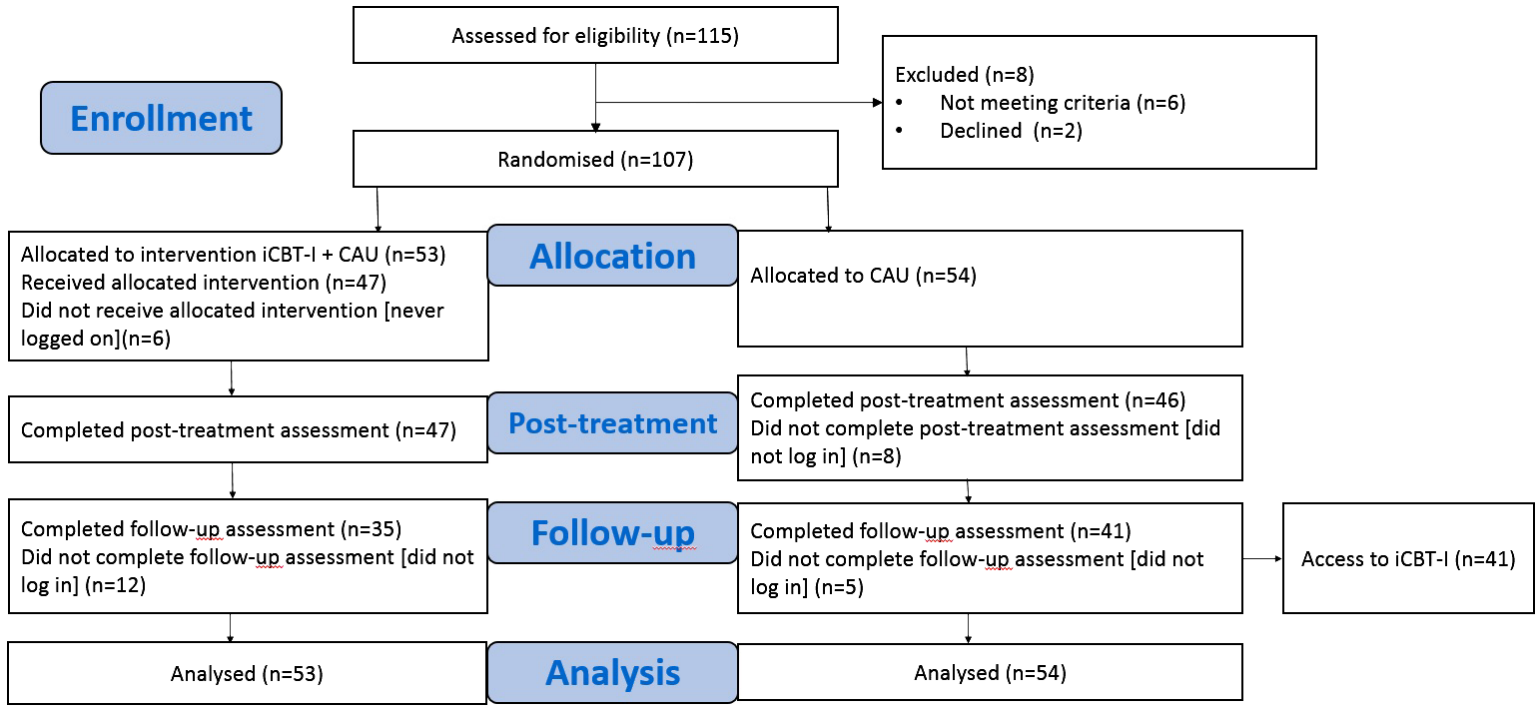
Participant flowchart. iCBT-I, internet-based cognitive behavioral therapy for insomnia; CAU, care as usual group.

The patients independently familiarized themselves with all the materials. They received feedback about their progress in sleep statistics over time from an expert in sleep medicine weekly via email, and were encouraged to consult her if they faced questions on how to adjust CBT-I techniques to their situations. Assessments were made at weeks 0 (t0, baseline), week 8 (t1, post-treatment), and week 20 (t2, follow-up) for both groups. The control group participants who completed the follow-up assessment were subsequently offered the iCBT-I program and assessed additionally after completion of the program at week 28 (t3, post-treatment control group). In the current analysis, we used data at the time points t0, t1, and t2. The participant flowchart is presented in **Figure 1**.

### 2.1 Participants

In total, 115 patients with CI fulfilling ICSD-3 criteria referred by neurologists specialized in sleep medicine in study clinical centers—Sleep Medicine Department, University Clinic 3, Sechenov First Moscow State Medical University, Moscow (*n* = 109) accepting patients from Moscow and other regions of the country; Stavropol Regional Clinical Sleep Center (*n* = 3); and Kuzbass Clinical Hospital for veterans (*n* = 3)—from March 2020 to December 2022 were assessed for eligibility. Eight patients were excluded due to the following reasons: comorbid sleep disorders (severe obstructive sleep apnea syndrome, poor internet knowledge, and exceeding the cutoff score of the BAI and BDI). The remaining 107 patients were considered eligible and signed an informed consent form. Since one part of the recruitment phase was conducted during the pandemic restrictions, some patients of the clinic were not able to see a doctor in person and were recruited through clinical interviews by phone. In both the in-clinic and phone interview insomnia criteria, concomitant sleep, psychiatric diseases, skills in using the internet, BAI, and BDI scores were assessed and used to check inclusion and exclusion criteria. The informed consent forms in these cases were signed by the participant and a researcher and sent by mail.

### 2.2 Primary and secondary outcomes

The primary outcome of the present study was the severity of insomnia measured with the Insomnia Severity Index (ISI) yielding a total score ranging from 0 (no insomnia) to 28 (severe insomnia). This is an assessment tool that examines both the nighttime and daytime aspects of insomnia disorder and is sensitive to insomnia severity change after CBT-I (25–27). Anxiety and depressive symptoms were assessed with the BAI and the BDI (28–30), comprising 21 questions and yielding a total score ranging from 0 to 63. A BAI total score higher than 25 corresponds to severe anxiety, and a BDI cutoff higher than 28 indicates severe depression. The health-related quality of life was measured by Short-Form Survey (SF-12) version 1.0 where higher scores indicate better health quality (31, 32). Common daytime symptoms of insomnia represented by the sense of fatigue and sleepiness were assessed with the Fatigue Severity Scale (FSS) (33) and Epworth Sleepiness Scale (ESS) (34) with higher scores indicating greater manifestation of the symptoms. Perpetuating factors of insomnia reflected in sleep habits and sleep-related beliefs were evaluated by the Sleep Hygiene Index (SHI) (35), Sleep Locus of Control Questionnaire (SLC) (27, 36), and Dysfunctional Beliefs and Attitudes About Sleep Scale (DBAS) (27, 37) accordingly. A high score on the questionnaires corresponds to a greater intensity of the factor. Forward and backward translation for the questionnaires was conducted if their validated version in Russian was not available.

Week average subjective sleep characteristics were derived from the consensus sleep diary capturing SE, TST, sleep onset latency (SOL), wake time after sleep onset (WASO), number of awakenings, and sleep quality (38). Two or more completed sleep diaries in a given week were required for the calculation of the weekly average subjective sleep characteristics; otherwise, the average subjective sleep parameters were set to missing for that particular week. Participants were filling in all the questionnaires and sleep diaries online at the Qualtrics Survey platform under their individual code. They received automatic reminders about the assessment every week for 1 month until they had completed the assessment. Adherence to the iCBT-I program was evaluated by the number of completed modules, the number of completed sleep diaries in the iCBT-I program, and the number of emails sent to the iCBT-I specialist during the program (excluding the emails about technical issues with the program). Participants were encouraged to ask questions and discuss arising problems in response emails; thus, the number of emails was not limited. Owing to technical reasons, it was not possible to evaluate such parameters as total time spent on the website, time spent on each module, and number of clicks.

### 2.3 Statistical analysis

To ensure 95% power to detect a small to medium between-group effect size (Cohen’s *d*) of 0.35 at post-treatment at an α level of 0.05, we calculated the sample size needed using a single-level repeated-measures ANOVA power analysis with G*Power (39), and our calculation yielded 55 participants per group and 110 for the total sample. The chosen effect size is reasonable since CAU being an active alternative treatment could decrease between-group outcome differences and a smaller effect size was considered to be irrelevant from a clinical point of view (16).

Linear mixed models in R were used to analyze the longitudinal data of the primary and secondary outcome measures at baseline, post-treatment, and after 3 months of follow-up. The approach of linear mixed models uses all available data of every subject without substituting missing values. Two models for the primary outcome were tested: (1) a base model testing the change of insomnia severity in all individuals across the three measurement time points; (2) the addition into the base model of the time*group interaction term (time*group) that shows the variability of the effect of time depending on the group. The models were tested using Akaike/Bayesian Information Criteria to select an optimal one. The best fit was for the model ISI_tg < −lmer (ISI ~ time*group + (1 | ID)) that included a time*group interaction term. The (1 | ID) term specifies that the intercept of the model varies randomly across individuals (ID), which assumes a random intercept model. In the final models, missing data were estimated using restricted maximum likelihood estimation. Within-group changes in outcome scores from posttreatment to follow-up were analyzed for the active condition. This was done with another mixed-effects model that included all time points and the respective outcome measure as the independent variable. Within- and between-group effect sizes (*d*s) were calculated based on estimated means from the mixed models using the following formula:


$$d=\;\frac{x_{1}-\;x_{2}}{s_{p}}$$

*x*_1_ and *x*_2_ are the means of the two groups at a certain time point or two time points being compared in between- and within-group analysis, accordingly; *s_p_* is the pooled standard deviation. We used the R package *lme4* to build linear mixed models and EMAtools to calculate Cohen’s *d*.

Furthermore, we conducted a sensitivity analysis to determine the robustness of our results for the primary and secondary outcomes. For this, we corrected models for the factors that could potentially influence the effect of therapy: any pharmacotherapy at baseline, and baseline scores of BAI and BDI. In order to assess the influence of adherence, we further conducted a per-protocol analysis. Only those participants of the iCBT-I + CAU group who had used the intervention for at least six out of eight modules were compared to all participants in the CAU group.

## 3 Results

Demographic characteristics and baseline clinical scores for the study sample are shown in **Table 1**. There was a significant difference in age and FSS between the two samples at baseline. Medications used for the management of insomnia included benzodiazepines and hypnotic benzodiazepine receptor agonists, sedating antidepressants, antipsychotics, antihistamines, melatonin, phytotherapy, and off-label substances. Ramelteon and orexin receptor antagonists were not used since they were not approved for use in Russia. The most frequently used medication group in both arms was antidepressants, then benzodiazepines or Z-drugs, followed by antipsychotics. There were no baseline between-group differences in the prevalence of use of all these medications. Only one of the enrolled participants had experience of the brief form of CBT-I before enrollment and none received any kind of CBT-I during the study period. Eight participants of the iCBT-I + CAU group and seven participants of the CAU group received regular psychological or psychotherapeutic help during the study.

**Table 1:** Baseline demographics and sample characteristics for intervention and control groups.

Characteristic	All (*n* = 107)	iCBT-I + CAU (*n* = 53)	CAU (*n* = 54)	Statistic
Age, years, median (IQR)	40 (31.5–53.0)	37 (28–50)	40.5 (34.2–60.5)	*U* = 1,105; *p* = 0.04*
Female, *n* (%)	62 (57.9)	30 (56.6)	32 (59.3)	*χ*^2^ = 0.78; *p* = 0.85
Duration of insomnia, months, median (IQR)	36 (17.75–79.5)	36 (18–66.5)	48 (12–84)	*U* = 1,154; *p* = 0.51
Use of medications for insomnia, *n* (%)	89 (82.4)	47 (88.7)	42 (77.8)	*χ*^2^ = 2.27; *p* = 0.20
Use of benzodiazepines and/or Z-drugs, *n* (%)	33 (30.8)	19 (35.8)	14 (25.9)	*χ*^2^ = 1.24; *p* = 0.30
Use of antidepressants, *n* (%)	42 (39.3)	24 (45.3)	18 (33.3)	*χ*^2^ = 1.40; *p* = 0.23
Use of antipsychotics, *n* (%)	21 (19.6)	11 (20.8)	10 (18.5)	*χ*^2^ = 0.06; *p* = 0.81
ISI, mean (SD)	15.4 (4.0)	15.4 (4.2)	15.4 (3.9)	*t* = −0.03; *p* = 0.97
BDI, mean (SD)	11.6 (6.2)	10.7 (5.7)	12.4 (6.5)	*t* = −1.47; *p* = 0.15
BAI, mean (SD)	9.5 (7.1)	8.7 (6.9)	10.3 (7.4)	*t* = −1.60; *p* = 0.25
SF-12, mean (SD)	31.4 (5.6)	31.7 (5.6)	31.1 (5.5)	*t* = 0.54; *p* = 0.59
DBAS, mean (SD)	105.6 (24.5)	101.2 (25.8)	109.8 (22.5)	*t* = −1.85; *p* = 0.07
LSC, mean (SD)	42.0 (12.9)	42.2 (11.8)	41.7 (14.0)	*t* = 0.19; *p* = 0.85
ESS, mean (SD)	5.2 (4.0)	4.8 (4.1)	5.5 (3.9)	*t* = −0.96; *p* = 0.34
SHI, mean (SD)	47.6 (7.5)	47.7 (8.2)	47.5 (6.8)	*t* = 0.14; *p* = 0.89
FSS, mean (SD)	39.8 (14.3)	36.2 (15.4)	43.2 (12.4)	*t* = −2.58; *p* = 0.01*
SE, %, mean (SD)	77.2 (12.1)	75.4 (12.8)	78.9 (11.1)	*t* = −1.43; *p* = 0.16
SOL, min, mean (SD)	43.0 (34.6)	40.7 (28.7)	45.2 (39.6)	*t* = −0.64; *p* = 0.52
WASO, min, mean (SD)	36.5 (39.4)	40.5 (43.3)	32.6 (35.2)	*t* = 0.99; *p* = 0.33
TST, hours, mean (SD)	6.7 (1.4)	6.7 (1.6)	6.8 (1.2)	*t* = −0.34; *p* = 0.10

ISI, Insomnia Severity Index; BDI, Beck Depression Inventory; BAI, Beck Anxiety Inventory; SF-12, Quality of Life Short-Form Survey; FSS, Fatigue Severity Scale; ESS, Epworth Sleepiness Scale; DBAS, Dysfunctional Beliefs about Sleep Scale; LCS, Locus Control of Sleep Scale; SHI, Sleep Hygiene Index; SE, sleep effectiveness; SOL, sleep onset latency; WASO, wake time after sleep onset; TST, total sleep time; iCBT-I + CAU intervention group; CAU, care as usual group; SD, standard deviation; IQR, interquartile range.

### 3.1 Dropout analysis

In total, 15 participants (14%)—7 from the iCBT-I + CAU group (13%) and 8 from the CAU group (15%)—did not complete the post-treatment assessment, although they were invited three times in weekly intervals via email. At follow-up, the number of dropouts was 31 (29%): 18 dropped out from the iCBT-I+CAU group (34%) and 13 dropped out from the CAU group (24%). In the intervention group, non-completers did not differ from completers. Lower dropout in the CAU group after follow-up was presumably due to the study design: these participants were motivated to receive access to the iCBT-I treatment following the completion of all questionnaires.

### 3.2 Main outcomes

For the primary outcome, at post-treatment, the linear mixed model for insomnia severity showed a significant time*group interaction effect, *F*(2, 178.96) = 6.03, *p* = 0.001, indicating that the change in ISI over time was significantly different between the two groups at post-treatment. Specifically, the estimated effect toward ISI decreased by 3.8-fold [standard error (SE) = 1.1] in the iCBT-I group compared to the CAU group. At follow-up, there were no significant between-group differences (*p* = 0.16).

Regarding secondary outcomes, at post-treatment, a significant time*group interaction effect to the advantage of the iCBT-I group was found in the model for DBAS, *F*(2, 175.21) = 3.85, *p* = 0.02; SE, *F*(2, 152.72) = 9.38, *p* < 0.001; and WASO, *F*(2, 151.66) = 4.34, *p* = 0.01. At follow-up assessment, the time*group interaction effect on DBAS and SE remained significant: *p* = 0.02 and *p* = 0.0001 accordingly, while the same effect on WASO was only nearly significant: *p* = 0.07. An interaction effect for TST was significant only after the follow-up: *p* = 0.02. The coefficients for the interaction term and effect sizes (Cohen’s *d*) are presented in **Table 2** for all outcomes.

**Table 2:** Estimated effect sizes for primary and secondary outcome measures and between-group effect sizes with confidence intervals in the iCBT-I + CAU group.

**Measure**	**Pretreatment, mean (SD)**	**Posttreatment, mean (SD)**	**Between-group effects at posttreatment**		**Follow-up, mean (SD)**	**Between-group effects at follow-up**	
			***B* (SE) *p***	***d* (95% CI)**		***B* (SE) *p***	***d* (95% CI)**
ISI iCBT-I + CAU CAU	15.4 (4.2) 15.4 (3.9)	10.0 (5.1) 13.5 (5.5)	3.8 (1.1) *p* = 0.001*	0.51 (1.66, 5.88)	11.3 (5.7) 13.1 (5.7)	1.7 (1.2) *p* = 0.16	0.21 (−0.63, 3.94)
BDI iCBT-I + CAU CAU	10.7 (5.7) 12.4 (6.5)	6.7 (5.2) 10.2 (5.4)	1.9 (1.1) *p* = 0.07.	0.27 (−0.15, 4,05)	8.0 (6.2) 10.3 (7.0)	0.8 (1.2) *p* = 0.51	0.10 (−1.50, 3.05)
BAI iCBT-I + CAU CAU	8.7 (6.9) 10.3 (7.4)	7.0 (6.4) 9.8 (7.7)	0.9 (1.3) *p* = 0.46	0.11 (−1.53, 3.40)	8.3 (7.9) 9.3 (7.6)	−0.6 (1.4) *p* = 0.68	−0.06 (−3.24, 2.10)
SF-12 iCBT-I + CAU CAU	31.7 (5.6) 31.1 (5.5)	34.7 (5.6) 33.1 (6.3)	−0.9 (1.1) *p* = 0.40	−0.13 (−3.07, 1.21)	33.7 (6.6) 32.8 (6.2)	−0.1 (1.2) *p* = 0.94	−0.01 (−2.39, 2.22)
FSS iCBT-I + CAU CAU	36.2 (15.4) 43.2 (12.4)	35.1 (14.0) 40.6 (13.8)	−1.5 (2.9) *p* = 0.61	−0.08 (−7.09, 4.15)	33.3 (14.9) 38.3 (15.2)	−2.4 (3.1) *p* = 0.45	−0.11 (−8.44, 3.71)
ESS iCBT-I + CAU CAU	4.8 (4.1) 5.5 (3.9)	4.4 (3.9) 4.1 (3.6)	−0.9 (0.6) *p* = 0.12	−0.24 (−2.12, 0.22)	3.9 (4.5) 4.0 (2.9)	−0.1 (0.7) *p* = 0.86	−0.03 (−1.39, 1.15)
DBAS iCBT-I + CAU CAU	101.2 (25.8) 109.8 (22.5)	75.3 (31.5) 95.8 (27.2)	11.8 (5.1) *p* = 0.02*.	0.35 (1.87, 21.74)	76.1 (33.6) 99.3 (27.7)	13.3 (5.5) *p* = 0.02*	0.36 (2.52, 24.03)
LCS iCBT-I + CAU CAU	42.2 (11.8) 41.7 (14.0)	38.5 (9.6) 38.6 (9.9)	0.6 (2.3) *p* = 0.81	0.04 (−3.97, 5.12)	39.8 (12.0) 40.1 (13.6)	1.6 (2.5) *p* = 0.52	0.10 (−3.31, 6.52)
SHI iCBT-I + CAU CAU	47.7 (8.2) 47.5 (6.8)	51.9 (7.2) 49.8 (6.5)	−2.2 (2.0) *p* = 0.28	−0.13 (−6.16, 1.73)	27.7 (7.7) 28.5 (6.3)	0.8 (2.2) *p* = 0.70	0.05 (−3.36, 5.03)
SE iCBT-I + CAU CAU	75.4 (12.8) 78.9 (11.1)	84.5 (10.4) 81.9 (9.9)	−6.3 (2.0) *p* = 0.002*	−0.51 (−10.17, −2.41)	83.3 (8.2) 80.2 (11.9)	−9.7 (2.5) *p* = 0.0001*	−0.63 (−14.50, −4.94)
SOL iCBT-I + CAU CAU	40.7 (28.7) 45.2 (39.6)	28.2 (29.0) 39.2 (37.2)	4.4 (7.1) *p* = 0.54	0.10 (−8.86, 18.65)	33.3 (28.2) 42.5 (37.1)	5.8 (8.7) *p* = 0.51	0.10 (−10.89, 22.83)
WASO iCBT-I + CAU CAU	40.5 (43.3) 32.6 (35.2)	19.0 (16.7) 28.0 (35.2)	18.5 (6.6) *p* = 0.01*	0.47 (5.78, 31.03)	28.8 (22.0) 29.3 (29.3)	15.1 (8.1) *p* = 0.07.	0.30 (−0.72, 30.74)
TST iCBT-I + CAU CAU	6.7 (1.6) 6.8 (1.2	6.9 (1.3) 7.0 (1.2)	−0.1 (0.2) *p* = 0.67	−0.07 (−0.52, 0.35)	7.3 (1.3) 7.0 (1.1)	−0.6 (0.3) *p* = 0.02*	−0.38 (−1.16, −0.09)

ISI, Insomnia Severity Index; BDI, Beck Depression Inventory; BAI, Beck Anxiety Inventory; SF-12, Quality of Life Short-Form Survey; FSS, Fatigue Severity Scale; ESS, Epworth Sleepiness Scale; DBAS, Dysfunctional Beliefs about Sleep Scale; LCS, Locus Control of Sleep Scale; SHI, Sleep Hygiene Index; SE, sleep effectiveness; SOL, sleep onset latency; WASO, wake time after sleep onset; TST, total sleep time; iCBT-I, internet-based cognitive behavioral therapy for insomnia; CAU, care as usual group; B (SE), estimated mean difference (standard error); CI, confidence interval; IQR, interquartile range. * statistically significant.

In the within-group analysis, we observed a significant decrease of ISI from pre- to post-treatment in the intervention group, −5.7 (SE = 0.8), *p* < 0.0001, and from pre-treatment to follow-up, −4.1 (SE = 0.9), *p* < 0.0001, *F*(2, 88.8) =27.2. Participants from CAU also significantly improved although to a lesser extent, −1.9 (0.7), *p* = 0.01 at post-treatment and −2.5 (0.8), *p* = 0.001 at follow-up, *F*(2, 90.2) = 5.9. Significant effect of time at both post-treatment and follow-up was observed for BDI, DBAS, SHI, SE, WASO, LCS, and SOL in the intervention group, *F*(2, 73.5–95.0) = 10.4–107.1, all *p*s < 0.001. In the CAU group, we observed significant improvement for BDI, ESS, DBAS, LCS, and SE at post-treatment. At follow-up, BDI, DBAS, SHI, SE, WASO, and TST significantly decreased in the intervention group. In the CAU group, significant improvement at follow-up was observed for BDI, FSS, ESS, DBAS, and SHI. See **Tables 3**, **4** for the estimated means and effect sizes of within-group analysis for all outcomes in the iCBT-I + CAU and CAU groups.

**Table 3:** Estimated effect sizes for primary and secondary outcome measures and within-group effect sizes with confidence intervals in the iCBT-I + CAU group.

Measure	Pretreatment, mean (SD)	Posttreatment, mean (SD)	Within-group effects at posttreatment		Follow-up, mean (SD)	Within-group effects at follow-up	
			*B* (SE) *p*	*d* (95% CI)		*B* (SE) *p*	*d* (95% CI)
ISI	15.4 (4.2)	10.2 (5.1)	−5.7 (0.8) *p* < 0.0001 *	−1.52 (−7.23, −4.13)	11.3 (5.7)	−4.1 (0.9) *p* < 0.0001 *	−0.96 (−5.84, 2.38)
BDI	10.7 (5.7)	6.6 (5.4)	−3.9 (0.8) *p* < 0.0001 *	−1.06 (−5.52, −2.39)	8.0 (6.2)	−2.7 (0.9) *p* = 0.004*	−0.63 (−4.43, −0.93)
BAI	8.7 (6.9)	7.1 (6.4)	−1.6 (0.9) *p* = 0.09	−0.37 (−3.32, −0.21)	8.3 (7.9)	−0.2 (1.0) *p* = 0.81	−0.05 (−2.22, 1.73)
SF-12	31.7 (5.6)	34.7 (5.6)	2.9 (0.8) *p* = 0.0004*	0.80 (1.36, 4.41)	33.7 (6.6)	1.9 (0.9) *p* = 0.03*	0.46 (0.20, 3.59)
FSS	36.2 (15.4)	34.7 (13.6)	−1.0 (2.1) *р* = 0.63	−0.10 (−5.10, −3.09)	33.3 (14.9)	−2.0 (2.3) *р* = 0.39	− 0.18 (−6.62, −2.57)
ESS	4.8 (4.1)	4.5 (4.0)	−0.3 (0.5) *р* = 0.53	−0.14 (−1.17, 0.59)	3.9 (4.5)	−1.0 (0.5) *р* = 0.05	−0.43 (−2.00, −0.02)
DBAS	101.2 (25.8)	76.1 (30.5)	−26.1 (4.0) *p* < 0.0001 *	−1.40 (−33.93, −18.35)	76.1 (33.6)	−23.9 (4.5) *p* < 0.0001 *	−1.13 (−32.71, −15.23)
LCS	42.2 (11.8)	38.3 (9.8)	−4.0 (1.6). *р* = 0.02*	−0.53 (−7.17, −0.83)	39.8 (12.0)	−2.7 (1.8) *р* = 0.15	−0.31 (−6.20, 0.90)
SHI	47.7 (8.2)	51.6 (7.4)	4.6 (1.5) *р* = 0.003*	0.51 (1.58, 7.61)	27.7 (7.7)	−20.0 (1.7) *p* < 0.0001 *	−2.0 (−23.08, −16.49)
SE	75.4 (12.8)	84.5 (10.4)	9.2 (1.5) *p* < 0.0001 *	1.48 (6.32, 12.16)	83.3 (8.2)	10.6 (2.0) *p* < 0.0001 *	1.19 (6.57, 14.57)
SOL	40.7 (28.7)	28.2 (29.0)	−12.9 (4.1) *p* = 0.003*	−0.75 (−20.96, −4.77)	33.3 (28.2)	−9.0 (5.6) *p* = 0.11	−0.36 (−19.92, 1.97)
WASO	40.5 (43.3)	19.0 (16.7)	−21.4 (4.8) *p* < 0.0001 *	−1.06 (−30.79, −11.98)	28.8 (22.0)	−16.7 (6.6) *p* = 0.01*	−0.56 (−29.56, −3.63)
TST	6.7 (1.6)	6.9 (1.3)	0.3 (0.2) *p* = 0.06	0.49 (−0.01, 0.66)	7.3 (1.3)	0.9 (0.2) *p* = 0.0003*	0.89 (0.42, 1.33)

ISI, Insomnia Severity Index; BDI, Beck Depression Inventory; BAI, Beck Anxiety Inventory; SF-12, Quality of Life Short-Form Survey; FSS, Fatigue Severity Scale; ESS, Epworth Sleepiness Scale; DBAS, Dysfunctional Beliefs about Sleep Scale; LCS, Locus Control of Sleep Scale; SHI, Sleep Hygiene Index; SE, sleep effectiveness; SOL, sleep onset latency; WASO, wake time after sleep onset; TST, total sleep time; iCBT-I, internet-based cognitive behavioral therapy for insomnia; CAU, care as usual group; B (SE), estimated mean difference (standard error); CI, confidence interval; IQR, interquartile range. * statistically significant.

**Table 4:** Estimated effect sizes for primary and secondary outcome measures and within-group effect sizes with confidence intervals in the CAU group.

Measure	Pretreatment, mean (SD)	Posttreatment, mean (SD)	Within-group effects at posttreatment		Follow-up, mean (SD)	Within-group effects at follow-up	
			*B* (SE) *p*	*d* (95% CI)		*B* (SE) p	*d* (95% CI)
ISI	15.4 (3.9)	13.5 (5.5)	−1.9 (0.7) *p* = 0.01 *	−0.54 (−3.38, −0.47)	13.1 (5.7)	−2.5 (0.8) *p* = 0.001 *	−0.66 (−3.98, −0.95)
BDI	12.4 (6.5)	10.2 (5.4)	−1.9 (0.7) *p* < 0.001 *	−0.57 (−3.34, −0.52)	10.3 (7.0)	−1.8 (0.8) *p* = 0.02*	−0.52 (−3.31, −0.37)
BAI	10.3 (7.4)	9.8 (7.7)	−0.6 (0.9) *p* = 0.49	−0.15 (−2.4, 1.1)	9.3 (7.6)	−0.8 (0.9) *p* = 0.40	−0.18 (−2.62, 1.02)
FSS	43.2 (12.4)	40.6 (13.8)	−2.5 (2.0) *р* = 0.22	−0.26 (−6.37, −1.41)	38.3 (15.2)	−4.4 (2.1) *р* = 0.04*	−0.44 (−8.46, −0.35)
ESS	5.5 (3.9)	4.1 (3.6)	−1.2 (0.4) *р* = 0.003*	−0.14 (−2.01, −0.46)	4.0 (2.9)	−1.1 (0.4) *р* = 0.008*	−0.43 (−1.94, −0.32)
DBAS	109.8 (22.5)	95.8 (27.2)	−14.3 (3.2) *p* < 0.0001 *	−0.95 (−20.55, −8.04)	99.3 (27.7)	−10.6 (3.3) *p* < 0.002 *	−0.67 (−17.13, −4.09)
LCS	41.7 (14.0)	38.6 (9.9)	−3.5 (1.7). *р* = 0.04*	−0.43 (−6.73, −0.17)	40.1 (13.6)	−1.0 (1.7) *р* = 0.57	−0.12 (−4.43, 2.40)
SHI	47.5 (6.8)	49.8 (6.5)	2.4 (1.3) *р* = 0.07	0.51 (1.58, 7.61)	28.5 (6.3)	−19.0 (1.4) *p* < 0.0001 *	−2.0 (−23.08, −16.49)
SE	78.9 (11.1)	81.9 (9.9)	3.0 (1.3) *p* = 0.03 *	0.51 (0.39, 5.57)	80.2 (11.9)	1.1 (1.4) *p* = 0.47	0.16 (−1.76, 3.88)
SOL	45.2 (39.6)	39.2 (37.2)	−8.0 (5.7) *p* = 0.17	−0.32 (−19.15, 3.23)	42.5 (37.1)	−3.0 (6.2) *p* = 0.63	−0.11 (−15.24, 9.22)
WASO	32.6 (35.2)	28.0 (35.2)	−3.1 (4.4) *p* = 0.48	−0.16 (−11.61, 5.48)	29.3 (29.3)	−2.6 (4.8) *p* = 0.59	−0.12 (−11.99, 6.80)
TST	6.8 (1.2	7.0 (1.2)	0.2 (0.1) *p* = 0.09	0.39 (−0.04, 0.53)	7.0 (1.1)	0.3 (0.2) *p* = 0.12	0.36 (−0.06, 0.56)

ISI, Insomnia Severity Index; BDI, Beck Depression Inventory; BAI, Beck Anxiety Inventory; SF-12, Quality of Life Short-Form Survey; FSS, Fatigue Severity Scale; ESS, Epworth Sleepiness Scale; DBAS, Dysfunctional Beliefs about Sleep Scale; LCS, Locus Control of Sleep Scale; SHI, Sleep Hygiene Index; SE, sleep effectiveness; SOL, sleep onset latency; WASO, wake time after sleep onset; TST, total sleep time; iCBT-I, internet-based cognitive behavioral therapy for insomnia; CAU, care as usual group; B (SE), estimated mean difference (standard error); CI, confidence interval; IQR, interquartile range. * statistically significant.

### 3.3 Effect sizes

Between- and within-group effect sizes (Cohen’s *d*) for different time points are presented in **Tables 2**, **3**. The between-group effect sizes on the ISI at post-treatment was *d* = 0.51; for iCBT-I vs. CAU in favor of the iCBT-I, it decreased to *d* = 0.21 at follow-up.

The between-group comparisons on the secondary outcomes at post-treatment revealed small to medium effect sizes in favor of the iCBT-I regarding DBAS (*d* = 0.35), SE (*d* = *−*0.51), and WASO (*d* = 0.47). At follow-up, the between-group effect size remained small to medium DBAS (*d* = 0.36), SE (*d* = 0.63), and TST (*d* = *−*0.38). Within-group comparisons of the primary and secondary outcomes revealed medium to large effect sizes on ISI, BDI, DBAS, LCS, SHI, SE, SOL, and WASO in the iCBT-I group at post-treatment (*d* = 0.51–1.52) and on ISI, BDI, DBAS, SHI, SE, WASO, and TST in the iCBT-I + CAU group after follow-up (*d* = 0.46–2.00).

### 3.4 Program usage

The average number of completed modules in the iCBT-I group was 6.14 out of eight modules, over the 8 weeks. The average number of the completed sleep diaries was 44.9 per participant, and on average, participants answered 8.7 emails from the guiding iCBT-I specialist.

### 3.5 Sensitivity analysis

In the sensitivity analysis, the adjustment for the current pharmacotherapy did not change the results for the primary outcome substantially. Correction of the model for baseline BAI and BDI scores did not change the effect size, but had better goodness of fit. The effect sizes of the adjusted models are presented in the **Supplementary Materials** (**Supplementary Table 1**). In the context of the sensitivity analysis, we also conducted per-protocol analyses of the main and secondary outcomes adjusted to the completion rate among iCBT-I group participants. We considered those who completed at least six modules (40 participants from 53) as completers because the first 6 modules covered all the behavioral and cognitive techniques, while the 7th and 8th modules are dedicated to the psychoeducation, repeat, and relapse plan. The per-protocol analysis showed similar effect sizes, albeit of a higher magnitude in most of the outcomes, and reaching statistical significance for BDI, *d* = 0.37 [95% CI (confidence interval): −0.40, 4.70]. Effect sizes of the models of the per-protocol analysis and models adjusted for the pharmacotherapy and for baseline BAI and BDI are presented in the **Supplementary Materials** (**Supplementary Table 1**).

## 4 Discussion

Recruitment from the clinical settings and CAU in both groups determines the high prevalence of concurrent pharmacotherapy in this study. Except for this, the baseline clinical characteristics and scores of our recruited patients were comparable with those in the previous studies of iCBT-I. The primary outcome was the significant reduction of insomnia severity in the iCBT-I group, which is in line with results of previous meta-analyses where iCBT-I has demonstrated comparable effect sizes ranging from moderate to large effects post-treatment in comparison with the control group (18, 40–42). As it was expected, the effect size is not large since a minor reduction of severity of insomnia symptoms could also be observed in the control group. However, even a medium effect is relevant for the investigated program, given that it is capable of reaching a lot of patients. The 95% CI, ranging from 1.66 to 5.88, lies above zero and corresponds to the meta-analysis by Seyffert M. et al. (2016) that supports the robustness of our results (18). The effect of sleep medications could mask the effect of nonpharmacological techniques, provided within the iCBT-I course, which can make patients less persistent and less motivated to follow recommendations after completion of active treatment. It should be noted that Hauri (1997) declared that the effect of CBT-I in combination with pharmacotherapy is less stable than that of CBT-I alone (43). However, the negative effect of sleep aids withdrawal on sleep outcomes was questioned by several authors (44, 45). Sensitivity analysis indicates that iCBT-I is equally effective in patients who took medications at baseline and those who did not receive treatment, i.e., medications did not reduce iCBT-I effects at post-treatment and follow-up.

By the follow-up assessment, the intervention group and the CAU group did not differ in insomnia severity. Meta-analysis of the long-term effects of face-to-face CBT-I has shown similar dynamics for ISI: between-group effect size gradually dropped from strong to moderate and small by 3, 6, and 12 months after the end of treatment (46). There was a similar dynamic of ISI in the intervention and control group in the study by Blom et al. (2016) (47). This scientific group also attributed this finding to the lesser consumption of pharmacotherapy, which was a part of the recommendation within the iCBT-I course in this study. However, our results appear inconclusive to the meta-analysis of Seyfert et al. (18), reporting the stability of the within-group effect of the iCBT-I. Stable within-group effect repeats the findings of Veda et al. (2019) and Luik et al. (2020) and the results of the meta-analysis by Zachariae et al. (2016) who reported robust within-group effects on main outcome after 12 and 18 months of follow-up (15, 48, 49).

The ICBT-I effect on dysfunctional beliefs about sleep was significant and robust in between-group analysis, which replicates the results of other iCBT-I studies (16, 50). In accordance with the previous studies, DBAS reinforces the sense of control in achieving better sleep and therefore can mediate the effects of CBT-I on the outcomes (50, 51). iCBT-I had a moderate between-group effect on SE post-treatment and after follow-up that was, in general, consistent with the previous studies demonstrating moderate to strong effects in research settings (18, 40) and in clinical settings (21).

The between-group effect of iCBT-I on SE and WASO was significant and ranged from small to moderate at post-treatment and follow-up. This is again consistent with the results of previous meta-analyses where the effect for WASO and SE ranged from moderate to large (18, 40, 42, 52). However, our findings contradict the results of Cheng et al. (2012) who reported no significant effect of the iCBT-I on WASO (41). As opposed to the literature, where iCBT-I has demonstrated significant between-group results for sleep onset latency, we did not find those in our study (40, 41). CAU as a control condition could be an explanation of this discrepancy. Most of the medications used by participants to control their sleep problems fell in the group of sleep aids acting on the GABA (γ aminobutyric acid) receptor–non-benzodiazepine Z-hypnotics. These medications are more likely to be effective in reducing sleep latency than in sleep maintenance (53, 54). Concerning the TST, the effects we found were larger than the results of iCBT-I described in the literature (18, 40, 41). Despite this, we may conclude that the effect of iCBT-I on the majority of the subjective sleep characteristics goes in line with the previous studies. The 95% CIs for the significant effects of the secondary outcomes (dysfunctional beliefs, SE, TSTs, and WASO) consistently align with the observed direction of effects—below or above zero, which strengthens the robustness of our findings.

The sensitivity analysis has shown that the baseline severity of depression and anxiety varying in a range determined by exclusion criteria does not change the effect of iCBT-I, which corresponds to the recommendations to use iCBT-I notwithstanding the presence of affective disorders (55). The absence of iCBT-I superiority over CAU in depression and anxiety symptom relief is inconsistent with the previous findings (40, 56). However, for the participants who completed six and more modules, iCBT-I has a significant small to moderate effect in alleviating depressive symptoms. This subgroup of patients has obtained an entire package of CBT-I techniques, especially cognitive techniques that were presented in modules 5 and 6, and an opportunity to practice them under the guidance of an iCBT-I specialist. This finding repeats the results of the meta-analysis where corrections for the adherence rate of 65% iCBT-I have shown greater effect sizes not only for depression, but also for anxiety and sleep outcomes (40).

One of the strengths of the current study is the recruitment in clinical settings, which allows us to generalize the results to patients in routine practice who may have insomnia comorbid with other medical or psychiatric conditions. Because of the well-determined inclusion and exclusion criteria, we were able to determine the target group more precisely and exclude other health problems that could severely affect the duration and depth of sleep. Dropout rate in our study was relatively low: 14% post-treatment and 29% after follow-up, which could be attributed to a higher motivation of patients to complete the procedure, since they were actively seeking treatment and referred to iCBT-I directly from the doctor’s office. Meta-analysis of randomized controlled trials of iCBT-I shows that an average dropout rate to follow-up is 24.7% in the iCBT-I group and 13.2% in the control group (15). The low dropout rate provided the study with sufficient power to detect a meaningful difference between groups after the treatment and at follow-up. The low dropout rate may indicate that participants recruited in the clinical settings are more motivated to complete the motivation and benefit from it.

There are, however, limitations of the present study to consider. The first is a rather short follow-up period. CAU, being the control condition, could reduce the power to detect a meaningful difference as was discussed previously. Owing to the nature of the intervention, blinding of participants was impossible, which increases the risk of bias (56). However, we assumed that the lack of blinding did not influence the study outcomes. It should be kept in mind that a smaller sample size can lead to less precise estimates, making them more susceptible to random variation or sampling error. The identified points limit the generalizability of our results and create the need for their verification in larger studies. The decision to opt for two diary entries per week for the calculation of weekly average values was undertaken to find a balance between mitigating data loss and ensuring enough representativeness of the average weekly values. However, employing only two diaries per week may compromise the accuracy of the weekly average sleep characteristics. Despite the limitations, the current study in sum provides evidence that in the clinical population under routine care conditions, iCBT-I was effective and showed similar effects although of somehow lower magnitude than in efficacy studies conducted in scientific settings. As a consequence, iCBT-I holds the potential to be effectively implemented in clinical settings and healthcare system and to contribute to alleviating the global burden associated with insomnia.

## 5 Conclusion

- iCBT-I decreases insomnia severity in comparison with CAU at post-treatment.
- iCBT-I decreases dysfunctional beliefs about sleep in comparison with CAU during the next 3 months after treatment.
- iCBT-I significantly decreases insomnia severity, depression symptoms, and dysfunctional beliefs about sleep; improves subjective sleep characteristics post-treatment and during the next 3 months after treatment; and improves sleep hygiene in iCBT-I patients during the next 3 months after treatment.

Despite its limitations, the present study extends the existing knowledge on iCBT-I effectiveness in clinical settings. iCBT-I has the advantage of accessibility to a large number of patients suffering from CI and can meet the existing need for mental health services.

## Data availability statement

The raw data supporting the conclusions of this article will be made available by the authors, without undue reservation.

## Ethics statement

The studies involving humans were approved by local ethics committee of the I.M. Sechenov Moscow Medical University. The studies were conducted in accordance with the local legislation and institutional requirements. The participants provided their written informed consent to participate in this study. Written informed consent was obtained from the individual(s) for the publication of any potentially identifiable images or data included in this article.

## Author contributions

PP: Conceptualization, Formal analysis, Investigation, Methodology, Writing – original draft. MP: Writing – review & editing, Investigation, Methodology. TK: Methodology, Writing – review & editing, Conceptualization. SD: Conceptualization, Methodology, Writing – review & editing. TB: Writing – review & editing, Supervision.
