# Supplementary material for: Clinical effectiveness of internet-based cognitive behavioral therapy for insomnia in routine secondary care: results of a randomized controlled trial
Source: Front Psychiatry. 2024 May 10;15:1301489. doi: 10.3389/fpsyt.2024.1301489 (PMC11116772; doi:10.3389/fpsyt.2024.1301489)
Supplement: Supplementary file 2 [file Table_1.docx]

Supplementary Table 1

|  | **Outcome ~ time*condition + (1 \| ID)** | | **Outcome ~ time*condition + adherence (1 \| ID)** | | **Outcome ~ time*condition + treatment_baseline (1 \| ID)** | | **Outcome ~ time*condition + BDI + BAI + (1 \| ID)** | |
| --- | --- | --- | --- | --- | --- | --- | --- | --- |
| **Measure** | **ES posttreat**  **B (SE), p**  **Cohen’s d, (95% CI)** | **ES FU**  **B (SE). p**  **Cohen’s d, (95% CI)** | **ES posttreat**  **B (SE). p**  **Cohen’s d, (95% CI)** | **ES FU**  **B (SE). p**  **Cohen’s d, (95% CI)** | **ES posttreat**  **B (SE). p**  **Cohen’s d, (95% CI)** | **ES FU**  **B (SE). p**  **Cohen’s d, (95% CI)** | **ES posttreat**  **B (SE). p**  **Cohen’s d, (95% CI)** | **ES FU**  **B (SE). p**  **Cohen’s d, (95% CI)** |
| ISI iCBT-I + CAU  CAU | **3.8 (1.1). p=0.001***  **0.51 (1.66. 5.88** | 1.7 (1.2). p=0.16  0.21 **(**-0.63. 3.94) | **4.2 (1.1). p=0.0003***  **-0.51 (1.99. 6.40)** | 2.1 (1.2). p=0.08  -0.26 **(**-0.24. 4.40) | **3.5 (1.1). p=0.001***  **0.48. (1.67. 5.90)** | 1.5 (1.2). p=0.19  0.19 (-0.61. 3.96) | **3.5 (1.1). p=0.001***  **0.49 (1.69. 5.91)** | 1.6 (1.2). p=0.15  0.21 (-0.48. 4.01) |
| BDI iCBT-I + CAU  CAU | 1.9 (1.1). p=0.07 .  0.27 (-0.15. 4.05) | 0.8 (1.2). p=0.51  0.10 (-1.50. 3.05) | **2.5 (1.1). p=0.02 ***  **0.37 (-0.40. 4.66)** | 0.8 (1.2). p=0.50  0.11 (-1.47. 3.02) | 1.8 (1.1). p=0.10  0.25 (-0.17. 4.03) | 0.7 (1.2). p=0.56  0.09 (-1.52. 3.03) | 1.8 (1.1). p=0.10  0.24. (-0.18. 3.99) ^1^ | 0.7 (1.2). p=0.55  0.09. (-1.49. 3.02) ^1^ |
| BAI iCBT-I + CAU  CAU | 0.9 (1.3). p=0.46  0.11 (-1.53. 3.40) | -0.6 (1.4). p=0.68  -0.06 (-3.24. 2.22) | 0.9 (1.3). p=0.51  0.11 (-1.68. 3.43) | -0.8 (1.4). p=0.55  -0.09 (-3.52. 1.88) | 0.9 (1.3). p=0.48  0.11 (-1.56, 3.38) | -0.6 (1.4). p=0.67  -0.06 (-3.27. 2.08) | 1.0 (1.3). p=0.44  0.12 (-1.46. 3.45) ^2^ | -0.5 (1.4). p=0.72  -0.05 (-3.15. 2.17) ^2^ |
| SF-12 | -0.9 (1.1) p=0.40  -0.13 (-3.07, 1.21) | -0.1 (1.2) p=0.94  -0.01 (-2.39, 2.22) | -1.3 (1.1), p=0.24  -0.19 (-3.55, 0.85) | 0.1 (1.2), p=0.91  0.02 (-2.17, 2.45) | -0.9 (1.1), p=0.41  -0.13 (-3.05, 1.23) | -0.1 (1.2), p=0.95  -0.01 (-2.38, 2.24) | -1.0 (1.1), p=0.36  -0.14 (-3.11, 1.10) | -0.3 (1.2), p=0.80  -0.04 (-2.56, 1.96) |
| FSS iCBT-I + CAU  CAU | -1.5 (2.9). p=0.61  -0.08 (-7.09. 4.15) | -2.4 (3.1). p=0.45  -0.11 ( -8.44. 3.71) | -0.3 (3.1). p=0.91  0.02 (-5.61. 6.27) | -2.4 (3.2). p=0.46  -0.11 (-8.62. 3.90) | -1.4 (2.9). p=0.64  -0.07 (-7.09. 4.15) | -2.3 (3.1). p=0.46  -0.10 (-8.44. 3.71) | -1.3 (2.9). p=0.64  -0.07. (-7.02. 4.17) | -2.0 (3.1). p=0.52  -0.09. (-8.03. 4.04) |
| ESS iCBT-I + CAU  CAU | -0.9 (0.6). p=0.12  -0.24 (-2.12. 0.22) | -0.1 (0.7). p=0.86  -0.03 (-1.39. 1.15) | -0.9 (0.6). p=0.13  -0.24 (-2.09. 0.27) | -0.2 (0.6). p=0.72  -0.06 (-1.48. 1.01) | -1.0 (0.6). p=0.12  -0.25 (-2.15. 0.18) | -0.2 (0.7). p=0.81  -0.06 (-1.43. 1.10) | -0.9 (0.6). p=0.12  -0.24. (-2.13. 0.21) | -0.1 (0.7). p=0.85  -0.03. (-1.40. 1.14) |
| DBAS iCBT-I + CAU  CAU | **11.8 (5.1). p=0.02*.**  **0.35 (1.87. 21.74)** | **13.3 (5.5). p=0.02***  **0.36, (2.52. 24.03)** | **13.7 (5.4). p=0.01*.**  **0.40 (3.28. 24.16)** | **14.4 (5.7). p=0.01***  **0.40 (3.40. 25.41)** | **11.3 (5.1). p=0.03***  **0.33 (2.07. 21.93)** | **13.1 (5.5). p=0.02***  **0.35 (2.73. 24.23)** | **11.2 (5.1). p=0.03***  **0.33 (1.97. 21.83)** | **13,2 (5,5), p=0,02***  **0,36 (2.77, 24.26)** |
| LCS iCBT-I + CAU  CAU | 0.6 (2.3). p=0.81  0.04 (-3.97. 5.12) | 1.6 (2.5). p=0.52  0.10 (-3.31. 6.52) | 1.1 (2.4). p=0.65  0.07 (-3.58. 5.75) | 2.5 (2.5). p=0.33  0.16 (-2.44. 7.40) | 0.3 (2.4). p=0.90  0.02 (-4.02. 5.07) | 1.4 (2.5). p=0.57  0.08 (-3.37. 6.47) | 0.3 (2.4). p=0.90  0.02. (-4.02. 5.06) | 1,4 (2,5), p=0,57  0,08, (-3.36, 6.47) |
| SHI iCBT-I + CAU  CAU | -2.2 (2.0). p=0.28  -0.13, -6.16. 1.73 | 0.8 (2.2). p=0.70  0.05 (-3.36. 5.03) | -2.6 (2.2). p=0.24  -0.13 (-6.80. 1.66) | 1.5 (2.3). p=0.52  0.08 (-2.95. 5.86) | -1.9 (2.0). p=0.35  -0.11 (-6.17. 1.72) | 1.0 (2.2). p=0.63  0.06 (-3.37. 5.02) | -1.9 (2.0). p=0.35  -0.11. (-6.14. 1.68) | 1,0 (2,1), p=0,65  0,06, (-3.38, 4.94) |
| SE iCBT-I + CAU  CAU | **-6.2 (2.0). p=0.002***  **-0.51 (-10.17. -2.41)** | **-9.60 (2.5). p=0.0002***  **-0.62 (-14.50. -4.94)** | **-6.4 (2.0). p=0.001***  **-0.56 (-10.26. -2.58)** | **-9.1 (2.4). p=0.0002***  **-0.64 (-13.84. -4.43)** | **-6.3 (2.0). p=0.002***  **-0.51 (-10.17. -2.41)** | **-9.7 (2.5). p=0.0001***  **-0.63 (-14.50. -4.95)** | **-6.3 (2.0). p=0.002***  **-0.52. (-10.19. -2.44)** | **-9,7 (2,5), p=0,0001***  **-0,63, (-14.47, -4.92)** |
| SOL iCBT-I + CAU  CAU | 4.7 (7.2). p=0.51  0.12 (-8.86. 18.65) | 6.3 (8.8). p=0.48  0.11 (-10.89. 22.83) | 6.4 (7.5). p=0.40  0.15 (-8.29. 21.05) | 5.4 (9.2). p=0.56  0.10 (-12.49. 23.26) | 4.6 (7.1). p=0.52  0.11. (-8.65. 18.86) | 5.3 (8.7). p=0.54  0.10. (-11.33. 22.35) | 4.5 (7.1). p=0.53  0.11 (-8.71. 18.77) | 5,9 (8,7), p=0,50  0,11, (-10.72, 22.97) |
| WASO iCBT-I + CAU  CAU | **18.1 (6.6). p=0.01***  **0.46 (5.78. 31.03)** | 16.1 (8.2). p=0.05.  0.32 (0.72. 30.74) | **21.0 (6.9). p=0.01***  **0.53 (7.65. 34.42)** | 15.7 (8.5). p=0.07.  0.31 (-0.95, 32.23) | **18.1 (6.6). p=0.01*1**  **0.47 (5.73. 30.94)** | **16.6 (8.2). p=0.04***  **0.30 (-0.44. 30.97)** | **18.6 (6.6). p=0.01***  **0.47. (5.78. 31.03)** | 15,2 (8,1), p=0,06  0,30 (-0.72, 30.74) |
| TST iCBT-I + CAU  CAU | -0,1 (0,2), p=0,71  -0,06 (-0.52, 0.35) | **-0,6 (0,3), p=0,03***  **-0,36 (-1.16, -0.09)** | -0,2 (0,2), p=0,45  -0,13 (-0.61, 0.26) | **-0,6 (0,3), p=0,03***  **0,39 (-1.16, -0.10)** | -0,1 (0,2), p=0,72  -0,06 (-0.51, 0.37) | **-0,6 (0,3), p=0,03***  **-0,38 (-1.17, -0.10)** | -0,1 (0,2), p=0,69  -0,07, (-0.52, 0.36) | **-0,6 (0,3), p=0,03***  **-0,37 (-1.15, -0.08)** |

Sensitivity analysis of models for primary and secondary outcome measures corrected for adherence to therapy, concurrent pharmacotherapy, and baseline anxiety and depression levels.

Abbreviations: ISI – insomnia severity index; BDI – Beck depression inventory; BAI - Beck anxiety inventory; SF-12 - quality of life Short-form survey; FSS – fatigue severity scale; ESS – epworth sleepiness scale; DBAS – dysfunctional beliefs about sleep scale; LCS – locus control of sleep scale; SHI – sleep hygiene index; SE – sleep effectiveness; SOL – sleep onset latency; WASO – wake after sleep onset; TST – total sleep time; iCBT-I + CAU intervention group; CAU – care as usual group; ; B (SE) – estimated mean difference (standard error); CI – confidence intervals; IQR – interquartile range

1 – in this models only BDI was added as a covariate, since BAI_t0 was icluded in the model as dependent variable chaning between timepoints

2 - in this models only BAI was added as a covariate, since BDI_t0 was icluded in the model as dependent variable chaning between timepoints
